# Global Research Trends and Hotspots Analysis of the Scientific Production of Amitriptyline: A Bibliometric Approach

**DOI:** 10.3390/ph16071047

**Published:** 2023-07-24

**Authors:** Cristian dos Santos Pereira, Jorddy Neves Cruz, Maria Karolina Martins Ferreira, Daiane Claydes Baia-da-Silva, Eneas Andrade Fontes-Junior, Rafael Rodrigues Lima

**Affiliations:** 1 Laboratory of Functional and Structural Biology, Institute of Biological Sciences, Federal University do Pará, Belém 66075-110, Brazil; chrisbiomed1008@gmail.com (C.d.S.P.); jorddy.cruz@icb.ufpa.br (J.N.C.);; 2 Laboratory of Pharmacology of Inflammation and Behavior, Federal University of Pará, Belém 66075-110, Brazil

**Keywords:** amitriptyline, therapeutic effect, bibliometric analysis, scientometrics

## Abstract

Amitriptyline was first introduced as a medication to treat depression. Over time, this substance has been used to treat other conditions, such as gastrointestinal disorders, fibromyalgia, neuropathic pain, and analgesia, among others. However, there are no published studies that provide a broad view of the possible motivations that have led to changes in the use of amitriptyline. In this study, we have identified the landscape of use for amitriptyline based on knowledge mapping of the 100 most-cited articles about this drug. We searched Web of Science Core Collection without time and language restrictions. We obtained 14,446 results, but we only used the 100 most-cited articles that had amitriptyline as the object of study. We collected the following information from each article: authors, country of the corresponding authors, year of publication, citation count, citation density (number of citations per year), and keywords. In addition, we seek to map in the chosen articles study design and research findings. We found that since 1980, the use of amitriptyline has expanded beyond depression, moving to off-label use to treat a variety of diseases and conditions, including post-herpetic neuralgia, neuropathic pain, primary fibrosis, fibromyalgia, and migraine, can be considered a drug with more clinical applicability than its original clinical indication.

## 1. Introduction

**Figure 1:**
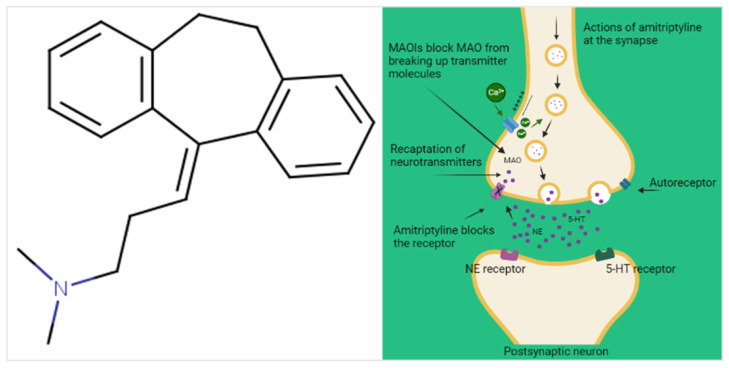
The molecular structure of amitriptyline and its mechanism of action in depression.

Amitriptyline [3-(10,11-dihydro-5Hdibenzo[a,d][7]annulen-5-ylidene)-N,N-dimethylpropan-1-amine], is a synthetic drug belonging to the class of tricyclic antidepressants (TCAs), characterized by the presence of three carbon rings in its structure (Figure 1). This drug is commonly used to treat depressive disorders and was introduced in the U.S. market in the early 1960s and is still used today, being referenced in the 2021 WHO List of Essential Medicines [1,2].

It is mainly used in the form of amitriptyline hydrochloride, appearing as water-soluble white crystals, orally administered, with high absorption in the gastrointestinal tract, providing an initial bioavailability of 90–95%. However, amitriptyline undergoes intense hepatic first-pass metabolism, which reduces its availability to about 53%. Its main hepatic metabolite, nortriptyline, retains similar pharmacological properties [3,4,5].

First pharmacodynamic evidence of amitriptyline activity reveals its ability to block serotonin and noradrenaline reuptake in adrenergic and serotonergic neuronal terminals, identified as the main mechanisms associated with its antidepressant effect. Activity on noradrenergic, serotonergic, muscarinic, histaminergic, and opioid receptors, among others, has also been described, often related to its sedative effect adverse reactions, and ‘off-label’ indications [3,6]. Despite being formally registered for depressive disorders pharmacotherapy, since the 1980s, amitriptyline began to be used for other clinical applications, with an emphasis on painful conditions such as neuropathic pain and fibromyalgia, in addition to non-organic nocturnal enuresis, and non-motor Parkinson’s symptoms, among many others [6,7].

Therefore, an important trend of transformation in the patterns of amitriptyline use can be seen, with possible repositioning in therapy, but the scientific literature still lacks evidence to support the long list of different clinical applicabilities in practice, especially in ‘off-label’ use. Thus, this study aims to map the most cited studies focused on amitriptyline and its applicability, highlighting the knowledge produced, the evolution of amitriptyline use, and the gaps that need to be filled.

## 2. Results

### 2.1. Selected Studies and Bibliometric Analysis

**Figure 2:**
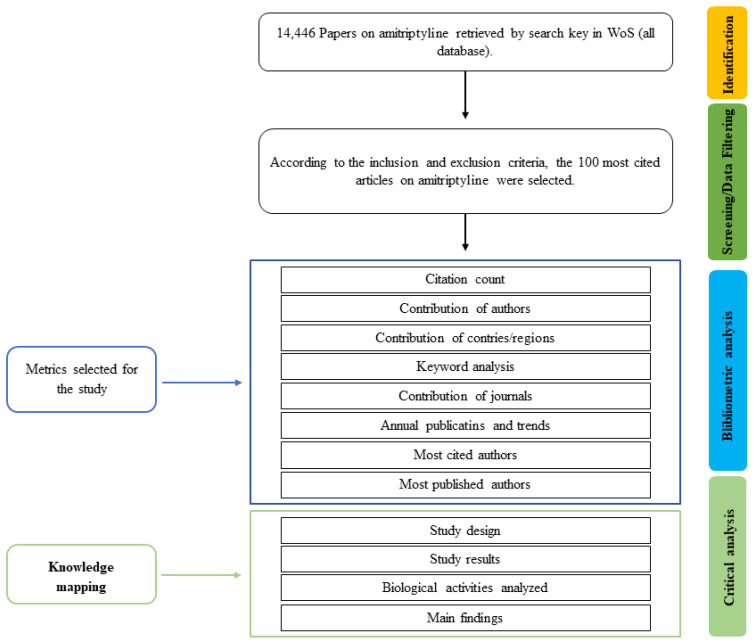
Flowchart of article screening.

Our search strategy produced 14,446 articles. After organizing the articles in descending order based on the number of citations, we evaluated a total of 189 articles. Of these, we selected 100 articles that met the eligibility criteria and excluded 89 because they did not meet the objective of this work (Figure 2).

The 100 selected articles were cited a total of 41,707 times. Authors A. Cipriani, R. Dubner, M.B. Max, B. Smoller, K. Fuxe, and E.S. Paykel published the most articles on the use of amitriptyline, with three articles each. The most cited article, among the 100 studies, was that by Steru et al., 1985 [8], entitled “The tail suspension test: a new method for scree of antidepressants in mice,” had been cited 2671 times. The least cited article was by Guyatt et al. entitled “The n-of-1 randomized controlled trial: clinical usefulness; since its publication in 1990, it has been cited 256 times. (Table 1).

#### 2.1.1. Publication Year

The selected articles were published between 1968 and 2018. We divided this range into 10-year periods and determined the number of papers for each period and the total number of citations that these articles received up to the time of data collection. Most of the articles were published between 1999 and 2008 (*n* = 39), which represents more than double the second period with more publications, however, this decade had the lowest citation density, with 384.21 citations per article (cit./art.). Articles published between 2009 and 2018 had the highest citation density, with 495.58 cit./art., also being the decade with the lowest number of articles published (Table 2).

#### 2.1.2. Journal of Publication

**Figure 3:**
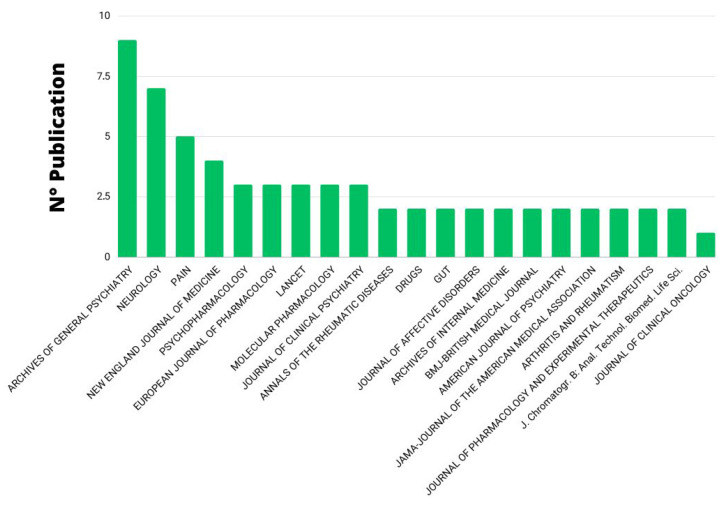
Journal of publication with at least two articles.

The 100 articles selected were published in 58 journals. The journal with more contributions was “Archives of general psychiatry” (*n* = 9; 2981 citations; 331.22 cit./art.) followed by “Neurology” (*n* = 7; 2671 citations; 381.57 cit./art.) and “Pain” (*n* = 5; 2051 citations; 410.2 cit./art.). The journal of publication most cited was “Psychopharmacology” (*n* = 3; 3230 citations; 1076.67 cit./art.) (Figure 3).

#### 2.1.3. Contributing Authors

Overall, there are a total of 493 authors among the 100 most-cited articles, with Steru, L, the most cited author in the Web of Science Core Collection (2671 citations). Cipriani, A.; Dubner, R.; Max, M.B.; Smoller, B.; Fuxe, K., and Paykel E.S. have published the most articles on the use of amitriptyline, with three articles each. In their study, Max, M.B. discussed the use of amitriptyline to treat diabetic neuropathy [10]. Cipriani A, and Paykel E.S., investigated the use of amitriptyline for depression [9,53]. Carlsson, A. and Fuxe, K. evaluated the ability of amitriptyline and other tertiary amines to block 5-hydroxytyramine uptake at central terminals [13].

The most cited study was by the author Steru, L. [8], with the title “The tail suspension test: a new method for the screening of antidepressants in mice” which had been cited 2671 times.

The second article with the most citations is by Cipriani, A., entitled “Comparative efficacy and acceptability of 21 antidepressant drugs for the acute treatment of adults with major depressive disorder: a systematic review and network meta-analysis” [9].

The least cited article among the top 100 studies was by author Lidbrink, P. [106] entitled “The effect of imipramine-like drugs and antihistamine drugs on uptake mechanisms in the central noradrenaline and 5-hydroxytryptamine neurons”, with a total of 245 citations. The author reports in his work that amitriptyline preferentially blocks 5-hydroxytryptamine receptors in central 5-hydroxytryptamine neurons.

**Figure 4:**
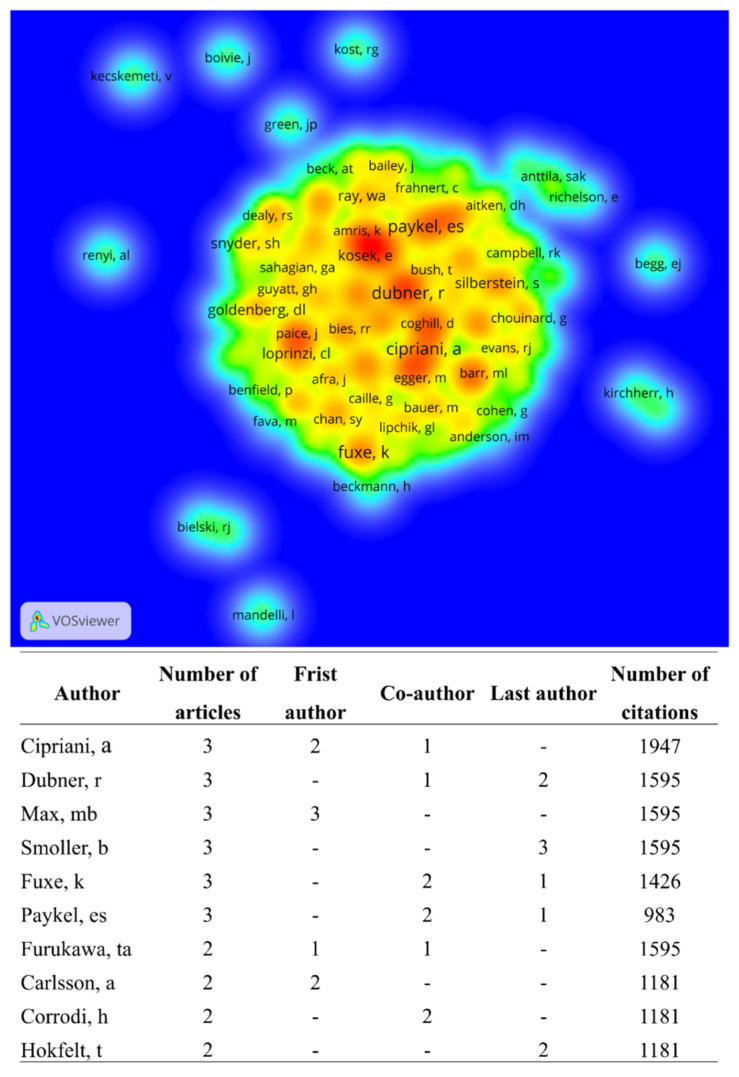
Visualization of authorship citation density. The colors indicate the author’s citation density, ranging from blue (lowest density) to red (highest density). The 493 authors formed 85 clusters. Authors with the highest number of published papers are identified, as are those with the highest number of citations, in order.

**Figure 5:**
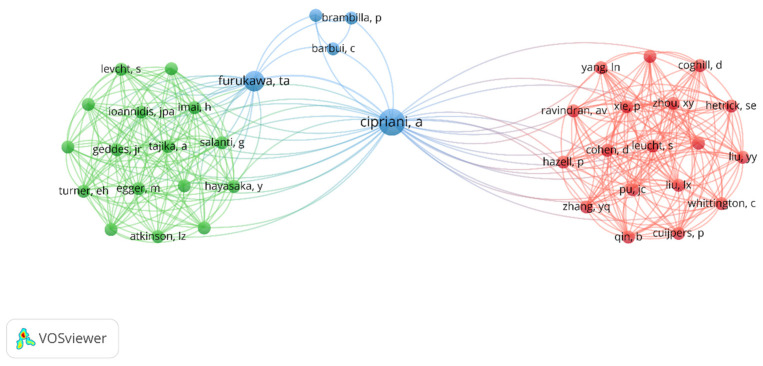
View of the network of author connections for the top 100 most cited articles on amitriptyline. The node size represents the number of publications. Nodes with the same colors represent the same cluster. Furthermore, the greater the link thickness and the smaller the distance between nodes, the greater the relative strength of the relationship. The colors are represented by blue, green, and red and interconnect the intensities of interactions.

We used VOSviewer to visualize the authorship citation density (Figure 4) and relationships between authors with the most links (Figure 5).

#### 2.1.4. Keyword Network

**Figure 6:**
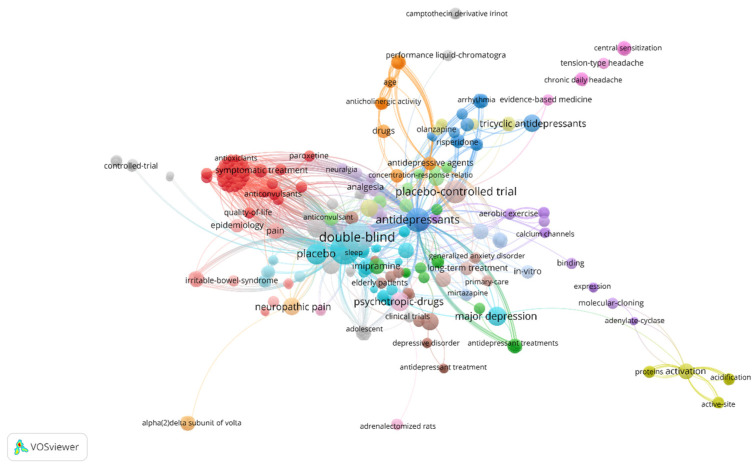
Keyword network. The node size represents the keyword frequency. Nodes with the same color are part of the same cluster. The thicker the link and the smaller the distance between the nodes, the greater the relative strength of the relationship.

In the 100 most-cited articles, there were 556 author keywords, behind ‘Double-blind’ (*n* = 16) and ‘Amitriptyline’ (*n* = 16), followed by ‘antidepressants’ (*n* = 10) and ‘placebo-controlled’ trial (*n* = 9) the most used (Figure 6). As amitriptyline is an antidepressant, the word antidepressant is also prominent in the network. Most of the studies were double-blind randomized clinical trials that addressed the use of amitriptyline or compared the effectiveness of amitriptyline with other TCAs such as imipramine and selective serotonin reuptake inhibitor antidepressants such as fluoxetine. Because amitriptyline can be used to treat pain and neuropathic diabetes, those words are also present in the keyword network.

#### 2.1.5. Distribution Map of the 100 Most-Cited Articles

**Figure 7:**
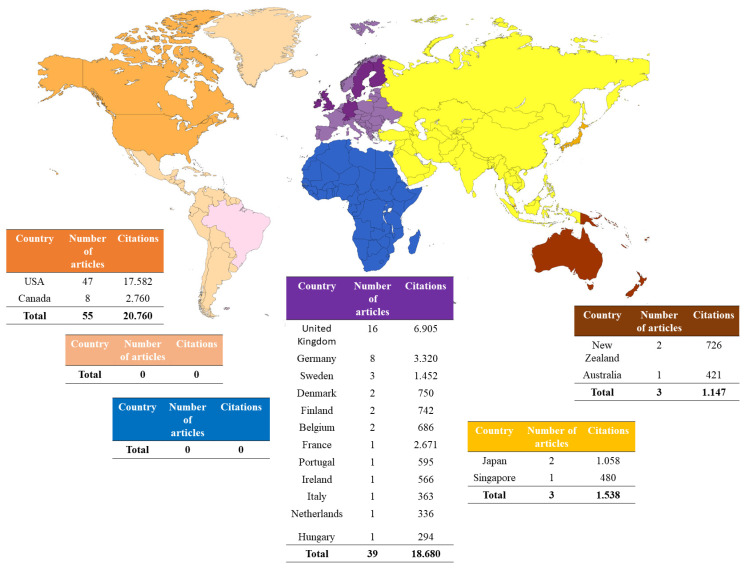
Worldwide distribution of the 100 most cited articles on amitriptyline.

Authors from North America, specifically the United States and Canada, are responsible for the majority of the 100 most-cited articles (*n* = 55), totaling 20,342 citations. Authors from European countries have also had a significant impact with 18,974 citations from 40 papers, with the most articles by authors from the United Kingdom (*n* = 16), Germany (*n* = 8), and Sweden (*n* = 3). On the other hand, there have been fewer articles from Asia and Oceania (Figure 7).

### 2.2. Content Analysis

#### 2.2.1. Study Design

Regarding study types, the literature reviews were the most frequent (*n* = 31; totaling 11,968 citations). Randomized clinical trials appear as the second most frequent type of study in the top 100 (*n* = 18; totaling 6537 citations); in vivo animal studies come right after randomized clinical trials (*n* = 16; with 8116 citations). Next come the systematic reviews, also with a significant number of studies (*n* = 13; with 6162 citations) (Table 3).

As previously demonstrated, the most cited article presents a murine model of depressive-like behavior, based on the tendency of rats to assume an immobility behavior when kept suspended by the tail for a prolonged period. The authors demonstrate that antidepressant drugs, including amitriptyline, reduce the immobility time of animals [107].

#### 2.2.2. Application of Amitriptyline

**Figure 8:**
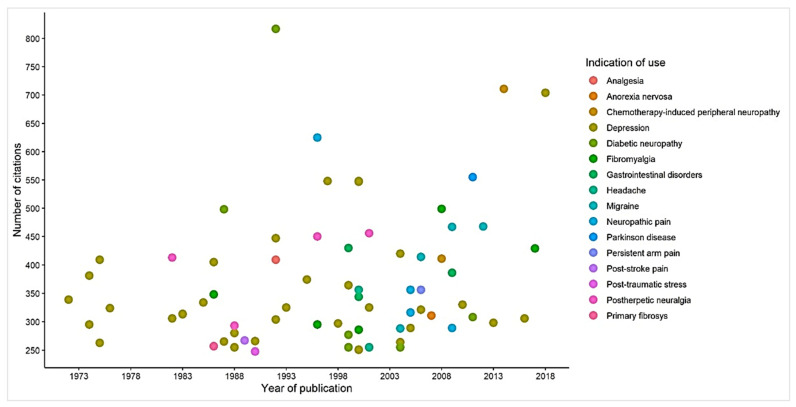
Knowledge mapping of the indication for the use of amitriptyline.

Of the most cited articles, 69 discussed the indication for the use of amitriptyline involving both human beings and animals, with 32 articles focusing on its official indication for depressive disorder and 37 on other indications not regulated by the registration bodies. Figure 8 shows the main indications over the years, from 1971 (the first article on use) to 2018 (the year of the last article).

**Figure 9:**
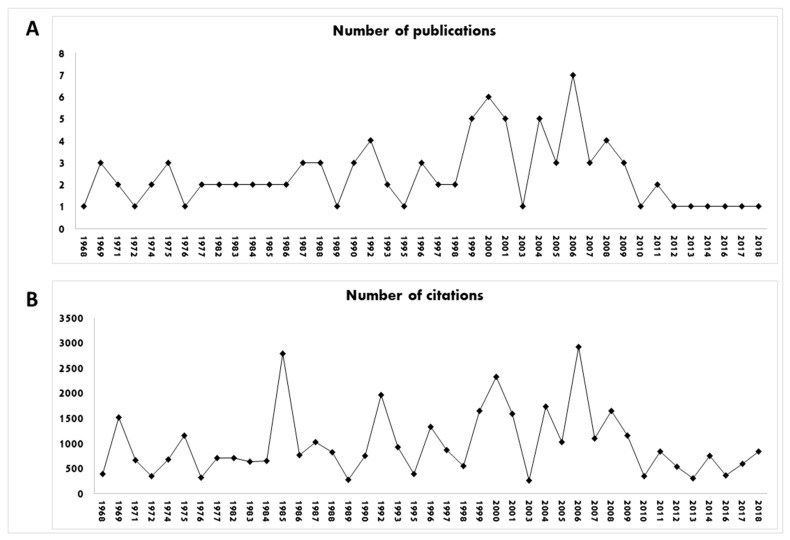
Number of publications (**A**) and number of citations (**B**) of the studies during the years. Years in which there were no publications were excluded.

Aiming to favor the analysis and interpretation of the central theme and relevance of the studies focused on the clinical application of amitriptyline, in Figure 9 we present the number of articles published in each year (Figure 9A), excluding the years in which there were no publications of selected articles, and the number of citations that the items received (Figure 9B).

The other 31 articles selected were aimed at elucidating pharmacokinetic or pharmacodynamic aspects of amitriptyline through in vitro techniques. In 1969, Ross et al. [61] reported that amitriptyline was more active than nortriptyline, its metabolite, in inhibiting 5-hydroxytryptamine uptake in brain tissue in an in vitro study. This study obtained a total of 336 citations in WoS (Core Collection).

The work of the author Ueng Y.F. (1997; 316 citations) is focused on the study of drug biotransformation mechanisms by P450 3A4, the most abundant P450 enzyme in the liver and small intestine and has an important role in amitriptyline biotransformation, as well as other drugs. In it, an in vitro P450 3A4 reconstitution system capable of reflecting the catalytic specificity of liver microsomes was used. The study demonstrates the cooperativity pattern in the oxidation of amitriptyline, testosterone, 17β-estradiol and, more notably, aflatoxin (FA) B1 and the influence of alpha-naphthoflavone in this process. However, no clear differences in patterns of reductase dependence of activities in relation to NADPH-P450 concentration have been reported with either AFB1, amitriptyline, or 17β-estradiol as a substrate [67].

Another of these studies that did not have a clinical intervention, is by the author Pancrazio, J.J., published in 1998, totaling 251 citations. Through the patch-clamp technique, applied to bovine adrenal chromaffin cells, this author was able to determine in vitro that TCAs, including amitriptyline, triggered effects on sodium (Na+) channels similar to local anesthetic behavior, a mechanism that may contribute to understanding analgesic properties [105].

##### Studies In Vivo

In the preclinical studies included, the oldest study was published in 1969, which investigated the antidepressant effects of amitriptyline, and the most recent among this type of study was published in 2008, where the anticholinergic action of several drugs was evaluated, including amitriptyline. Overall, we retrieved 15 in vivo studies, and it is possible to note that the greatest research interest is in antidepressant effects (*n* = 9). However, we can point out that other objects of the investigation were also discussed, such as diabetic neuropathy (*n* = 1), cystic fibrosis (*n* = 1), migraine (*n* = 1), anticholinergic investigation (*n* = 1), inhibition, and catecholamine uptake (*n* = 2).

Among these in vivo studies, the most cited (*n* = 2671) was that of the author Steru et al. (1985), with the theme entitled “The tail suspension test—a new method for antidepressant screening in mice”. In this work, the author used a method that places the mouse suspended by the tail to test antidepressants by recording its movements. This test had a total duration of 6 min, divided into periods of immobility and agitation. As a result of this study, it was possible to observe that antidepressants, including amitriptyline, are able to decrease the duration of immobility in mice [8].

The second most cited (722) article among in vivo animal studies corresponds to the author Carlsson et al. (1969), entitled “Effect of antidepressant drugs on depletion of intraneuronal brain 5-hydroxytryptamine stores caused by 4-methyl-alpha-ethyl-meta-tyramine”. This study used mice to inject 4-methyl-alpha-ethyl-meta-tyramine in two doses of 100mg/kg intraperitoneally. After a period of four hours, the animals were euthanized to remove their brains for later analysis. After performing the analyses, the author suggested that 5-HT reuptake blockade is probably involved in the mood-elevating action of tricyclic antidepressants, including amitriptyline. On the other hand, the author suggests that the blockade of noradrenaline reuptake promotes the impulse in the depressed patient [108].

The third most cited article (*n* = 484), “Studies on the distinction between uptake inhibition and release of [h-3] dopamine in rat brain tissue slices,” is authored by Heikkila et al. (1975). This document evaluated the confusion over the classification of drugs as uptake inhibitors or biogenic amine-releasing agents by analyzing rat brain slices (neostriatum and cortex). It was possible through this study to conclude that, in the tissue analysis, it was possible to observe a real release experimentally (action not materially affected by the reuptake blockade); on the other hand, a releasing action evoked an apparent inhibition of the uptake of magnitude equal to the releasing action of these agents inhibitors, including amitriptyline [27].

Among these studies, the least cited article (*n* = 259) was by Green et al. (1977), who published the article “Tricyclic antidepressant drugs block histamine h-2 receptor in the brain”, which used four tricyclic antidepressants, including amitriptyline, to be tested on the H2 receptor that is linked to adenylate cyclase in homogenates of the hippocampus and cortex of adult pigs. The author suggested that tricyclic antidepressants, including amitriptyline, exert competitive histamine H2 antagonist activity [101].

##### Randomized Clinical Trials, Non-Randomized and Systematic Review

A change in study subjects on the clinical use of amitriptyline has been observed over the years. From 1973 to 2018, a wide range of studies pointed to the use of this drug to treat conditions other than depression. The study published by Radley et al. [23], that received 522 citations, identified amitriptyline hydrochloride as the second drug with the highest proportion of off-label use, corresponding to 81% of the drug prescriptions evaluated, using US-FDA registration as a reference.

Randomized placebo-controlled clinical trials have demonstrated the efficacy of amitriptyline in treating painful conditions, including fibromyalgia and neuropathies, such as postherpetic, diabetic, or chemotherapy-related neuropathies, and chronic arm pain, among others. We also identified studies that investigated the application of amitriptyline for post-traumatic stress treatment.

**Figure 10:**
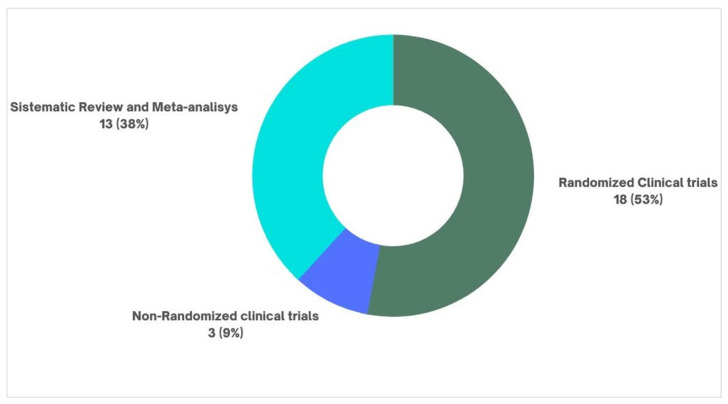
Clinical studies and systematic reviews.

Of this top 100, we had a total of 18 randomized clinical trials that had amitriptyline as the object of study, 13 systematic reviews, and three non-randomized clinical trials. We will highlight some of the most cited studies in the text and Figure 10.

Among selected articles, the first randomized clinical trial aimed at evaluating the amitriptyline use in a specific painful condition was published in 1982, with the theme “Amitriptyline versus placebo in postherpetic neuralgia”. In it, Watson, C.P. evaluated 24 patients who received amitriptyline (median dose = 75 mg) or placebo, identifying good to excellent pain relief in 16 of the 24 patients (*p* ≤ 0.001), not associated with an antidepressant effect. Thus, the author concluded that amitriptyline is useful in the treatment of PHN [51]. A second study focused on the treatment of PHN, authored by Max M.B., in 1988, evaluated 58 patients treated for 6 weeks with amitriptyline (12.5–150 mg/day), lorazepam (0.5–6 mg/day) or placebo. They identified pain relief in 47% of patients receiving amitriptyline versus 16% in the placebo group. Greater relief was identified at higher doses [82].

In the article “A randomized, controlled trial of amitriptyline and naproxen in the treatment of patients with fibromyalgia”, published in 1986, receiving 354 citations, Goldenberg et al., followed 62 patients, divided into 4 groups, comparing 6 weeks of treatment with amitriptyline, naproxen, or amitriptyline + naproxen with a group that received placebo. Amitriptyline (25 mg/kg at night) alone promoted significant improvement in pain, sleep difficulty, fatigue on awakening and tender points score [58].

The study titled “Effects of Desipramine, Amitriptyline, and Fluoxetine on Pain in Diabetic Neuropathy” (1987) by author Max, M.B. compared amitriptyline (12.5 to 150 mg/day) with other drugs in the treatment of painful diabetic neuropathy. This study was performed on 84 patients, who were separated, allowing comparison between drugs. 74% of patients treated with amitriptyline experienced moderate to complete pain relief, significantly greater than placebo, regardless of the presence of depression [25].

In one randomized clinical trial, 46 veterans with chronic post-traumatic stress disorder were treated with amitriptyline or placebo for 8 weeks. The best results were seen on the Hamilton Depression Scale at 4 and 8 weeks, where amitriptyline was superior to placebo. However, there was no evidence of an effect based on the interview to diagnose post-traumatic stress disorder [104].

Therefore, although the study on the use of amitriptyline to treat depressive disorders concentrates the largest number of publications, in the selected top 100, we found more randomized clinical studies exploring other applications of the drug.

Regarding systematic reviews, the most cited studies were by the authors Cipriani, A., and Hershman, D.L., followed by the author McQuay, H.J. The authors had 842, 789, and 636 citations, respectively. Cipriani, A., was the author with the highest number of citations among the top 100 systematic reviews [54] with the theme entitled “Comparative effectiveness and acceptability of 21 antidepressant drugs for the acute treatment of adults with major depressive disorder: a systematic review and network meta-analysis”, with a total of 842 citations. The author described that antidepressants, including amitriptyline, were more effective than a placebo in adults with major depressive disorder.

Hershman D.L., author of the work “Prevention and Management of Chemotherapy-Imposed Peripheral Neuropathy in Survivors of Adult Cancers: American Society of Clinical Oncology Practice Guideline”, published in 2014 and totaling 789 citations, obtained the second-highest number among systematic reviews. From the analysis of 48 randomized clinical trials (RCTs), which addressed the treatment of chemotherapy-induced peripheral neuropathy (CIPN) in adult cancer survivors, it listed recommended and not recommended drugs for prevention or treatment. It concluded that amitriptyline should not be indicated for the prevention of neuropathy. Regarding the treatment, he stated that there is no evidence of specific benefits, but that it could be used based on its effectiveness for other conditions of neuropathic pain [11].

In a similar vein, in the study “Systematic review of antidepressants in neuropathic pain”, published in 1996 by McQuay, H.J., which received 636 citations, 17 randomized controlled trials were reviewed that explored the safety and efficacy of the antidepressants used in the treatment of painful neuropathic conditions, such as diabetic neuropathy, atypical facial pain, and PHN. It identified an average pain relief of ~50%, with 30% of patients having minor adverse reactions and 4% having to interrupt treatment due to more severe reactions. The study concludes that antidepressants, including amitriptyline, are effective in the treatment of neuropathic pain when compared with placebo [15].

## 3. Discussion

Using bibliometric analysis, we have provided an overview of the scientific interest and trends in the use of amitriptyline over the years and the academic impact of these studies (based on the number of citations). The selected studies have different levels of scientific evidence. Most of them are literature review studies (*n* = 31) on the mechanisms of action of amitriptyline and the evolution of its indication for use (or clinical application). In addition, we identified studies such as randomized clinical trials (*n* = 18) and systematic reviews (*n* = 13), which, after literature reviews, appeared a lot in this top 100. We have noticed that this drug is no longer used exclusively to treat depression; it is also used off-label. Figure 8 shows that before 1978, this selection included only studies of amitriptyline for depressive disorders. After this period, until 2018, we noticed that studies emerged directing the use of amitriptyline for other diseases, such as neuropathic pain and fibromyalgia, for example. In addition, we also note that amitriptyline is no longer a first-choice drug to treat depression, with selective serotonin reuptake inhibitors having this role [109].

Citation counting is recognized as an effective method for measuring the scientific impact of articles, researchers, and journals, and is also used as a promoter of academic research and clinical practice. It is important, however, to rationally select the database to collect the number of citations, as each database adopts its own criteria for counting citations, generally related to journal indexing [110]. Previous studies report that the Web of Science rescues studies from the beginning of the 20th century through the Web of Science’s Science Citation Index Expanded [111]. According to Bakkalbasi et al. [112] and Falagas et al. [113], the WoS is one of the best tools available for citation analysis, due to the possibility of retrieving publications since 1945 and covering quality journals that publish articles from around the world. In this work, we performed the search including all WoS databases to obtain the 100 most-cited articles that dealt with amitriptyline. The success of the search is evidenced by a large number of articles with a high number of citations that we found.

Among the bibliometric findings, we identified that the authors with the highest number of published articles were also, for the most part, those with articles with the highest number of citations. The most cited article (2671 citations), by Steru et al. [8], in 1985, received 3.17 times more citations than the second most cited (842 citations), by Cipriani, published in 2018. Despite this, the second presented 2.4 times more citations each year of publication than the first, showing that publication time is not a determining factor for a greater citation. In addition, some authors with more than one article in this top 100 received a total number of citations lower than others who have only one article on the list. The author Cipriani A. has two articles [9,54] on the list, which together were cited 1207 times, 1464 fewer citations than the article by Steru L. [8]. Such observations confirm the relevance of the number of citations as an indicator of the impact of articles on scientific development.

Among the ten most cited articles, only four [9,11,16] are published in open-access journals, not being the case of the most cited article. In addition, the most cited articles are widely cited because they are freely available in several databases.

The high impact of Steru L. [8] can be explained by the fact that it presents a simple, practical, and relatively low-cost behavioral model, with a good degree of prediction for compounds with antidepressant potential, using a methodology of suspension by the tail of rats as a basis for the evaluation of drug candidates ever since.

We believe it is important to use the corresponding author’s country of origin as a criterion to delineate the geographical distribution of articles. Even recognizing that science is increasingly a global enterprise, integrating researchers from different regions of the planet in collaborative work, we consider that generally, the corresponding author assumes a leadership role in the working group, allowing the visualization of the regions and countries that led the engagement on this topic. We also verified that the United States gained a prominent place within the studies, concentrating 55% of the 100 selected studies, followed by the United Kingdom as the highlight of the European continent. This understanding is important for us to know where these studies are originating from and what contributions they are making to supply the scientific community with relevant information.

From the research, we found that amitriptyline, since its origin, has been registered in regulatory bodies, specifically for the treatment of depressive disorders, and is still found in reference bodies such as the US-FDA [3,114]. Naturally, the first published studies aimed to understand its mechanisms of action, pharmacokinetic characteristics, safety, and efficacy in the treatment of depression. However, it is also important to emphasize that amitriptyline is no longer a first-choice drug for depression, as it has lost this space to selective serotonin reuptake inhibitors [109].

It is already well established that amitriptyline is easily dissolved in an aqueous medium, with excellent gastrointestinal absorption and an intense first-pass effect in the liver, as well as its action in the central nervous system. However, ongoing investigations have also revealed its ability to act directly on receptors, such as adrenergic (alpha-1), histaminergic, and muscarinic (M1 and M3), which have been associated with side effects of its use, such as xerostomia and sedation, which shows its great importance and variety of actions in different places, which also facilitates the understanding of the therapeutic repositioning by this drug [3,6].

However, between the 1970s and 1980s, articles began to appear exploring the benefits of amitriptyline for the management of painful conditions, as seen in Figure 8, especially those associated with neuropathies, such as diabetic neuropathy, [25,82,89,90] and PHN [30], but not exclusively. We also looked at studies on the treatment of chronic tension headaches [50,97], migraines [21,26,32,88], and complex conditions such as fibromyalgia [16,24,58,78,86], among others. Non-painful conditions were also studied, such as fibrositis [102], gastrointestinal disorders [35,44,57], post-traumatic stress [104], and non-motor symptoms of Parkinson’s disease [17]. This transformation in the paradigms studied follows its use in clinical practice and seeks to subsidize the long list of uses currently classified as “off-label”, as they do not correspond to registration in official agencies [3]. We also observed that these studies were published in specific journals in the area, such as Pain, Psychopharmacology, and Psychiatry.

Considering the relative ineffectiveness of selective serotonin reuptake inhibitors in relieving neuropathic pain and the significant effect of antidepressants that inhibit noradrenaline reuptake, it is theorized that this latter is more relevant for analgesic activity, and may subsidize, at least in part, the action of amitriptyline [114]. In fact, intrathecal administration of α1-adrenergic agonists in rats induces analgesia, which may be related to the consequent increase in GABA release in the spinal cord. On the other hand, it is suggested that the NA elevation in the medulla dorsal horn can inhibit the synaptic transmission of the peripheral afferent fibers through the activation of pre-synaptic α2 receptors, Gi/0 protein-coupled receptors, which inhibit the cAMP synthesis and activate K+ hyperpolarizing currents. This mechanism seems to have a poor effect on triggering noxious stimuli but effectively controls hyperalgesia and allodynia linked to neuropathic pain. Increased NA levels also appear to normalize the descending inhibitory function of the Locus Coeruleos, which is impaired in persistent painful conditions associated with nerve damage, improving endogenous analgesia [99,114]. Other mechanisms that potentially contribute to the analgesic activity of amitriptyline are the inhibition of the neural reuptake of adenosine, increasing adenosine activity in the peripheral and central nervous systems [115]; blockade of Na+ channels, inhibiting ectopic discharges present in injured nerves; activation of opioid receptors, inhibition of nitric oxide synthesis, increased production of BDNF, among others [116].

Although the use as an antidepressant (15.928 citations) brings together the largest number of articles in our sample, if we group the articles that studied its use for other diseases (14.581 citations), that number is not surpassed. Currently, amitriptyline is still widely used for the treatment of depressive disorders, but it is generally left as a second or third choice in clinical protocols, due to its pattern of adverse reactions and greater chances of non-adherence. On the other hand, its use as an analgesic gained significant prominence, becoming a first-line drug for the treatment of neuropathic painful conditions and fibromyalgia [15,24,58,117].

This study may have limitations, as it does not guarantee a methodological evaluation of the selected articles, nor does it guarantee the certainty of the evidence. It presents an overview of the use of amitriptyline, provides more widespread information in the scientific community, and is not an instrument for clinical decision-making. For other proposals for the application of amitriptyline, the scenario can be challenging, given the scarcity or lack of studies that support the prescription for other diseases or conditions. We also wish to make clear some limitations on the use of research databases. We used Wos, SCOPUS, and Google Scholar to retrieve the citation number of some articles. However, only the Wos—Core Collection was used as a data source for information such as authors’ names, paper keywords, and corresponding author’s country of origin. WoS, SCOPUS, and Google Scholar have broad data coverage, with differences in coverage in certain languages (mainly) and some research areas. We selected the Web of Science Core Collection as a data source because it has a high index of indexed English documents in the area of Life Science and Biomedicine. Another point worth mentioning is that the database we use, has some limitations, such as not providing complete data on some open-access articles, and this database also belongs to a commercial provider that ends up demanding an access fee for access obtaining the studies in full [118]. Thus, more comprehensive studies are needed to support the use of amitriptyline. In this sense, our mapping should serve as a guide for future research on amitriptyline.

## 4. Materials and Methods

### 4.1. Data and Search Strategy

We employed the bibliometric method, which consists of selecting the most cited articles because the articles with the highest number of citations have had a great impact on the scientific community [112]. We searched the Web of Science Core Collection in March 2023, without restrictions of time and language, using the following search key topics described in the table below (Table 4). The Web of Science Core Collection includes Science Citation Index Expanded (SCIEXPANDED), Social Sciences Citation Index (SSCI), Arts & Humanities Citation Index (A&HCI), Conference Proceedings Citation Index-Science (CPCI-S), Conference Proceedings Citation Index-Social Science & Humanities (CPCI-SSH), Book Citation Index Science (BKCI-S), Book Citation Index Social Sciences & Humanities (BKCI-SSH), Emerging Sources Citation Index (ESCI), Current Chemical Reactions (CCR-EXPANDED), and Index Chemicus (IC) [118].

We selected the 100 most cited articles by organizing the results in descending order based on the number of citations. Two researchers (C.S.C. and J.N.C) carried out the selection independently; discrepancies in the selection of articles were resolved using the concordance method, steps already pre-established in the work by Nascimento et al. (2022) [119].

### 4.2. Eligibility Criteria

We included original research articles and review articles in which the pharmacological properties of amitriptyline had been investigated. We excluded all editorials and articles from conferences and studies that did not correspond to the specific theme. Studies, where amitriptyline was not the focus of investigation, were also excluded.

### 4.3. Extraction of Bibliometric Parameters

In an Excel file, available as a document extraction method in WoS itself, we collect the title, authors, year of publication, number of citations, citation density (number of citations per year), keywords, country, and continent of the author corresponding. We also performed searches in the Scopus and Google Scholar databases to compare the number of citations for selected articles. Google Scholar includes citations from books, free online journals, and non-academic sources in its database, in addition to offering a broader view of its content around the world, which generates more hits and consequently more citations. WoS and Scopus retrieve more citations from articles in peer-reviewed journals [112]. However, as these databases are commercial and subscription-based products, their use in the world results in a high price, which means that an institution is forced to choose one or the other [120].

We ranked articles according to the number of citations in the Web of Science Core Collection. In the event of a tie in the number of citations, we based the position of the citation density. We represented the country and continent results with maps created using an online tool (https://mapchart.net/ (accessed on 15 May 2023)).

### 4.4. Data Analysis

We analyzed the collected data and generated bibliometric networks regarding co-authorship and co-occurrences of all keywords by using the Visualization of Similarities Viewer software (VOSviewer 1.6.16) [121,122]. For the co-authorship map, we introduced the names of authors with at least one article into the software as a unit of analysis. We linked them together based on the number of articles of joint authorship. These results are shown in the network and density visualization. For the co-occurrence map, we introduced author keywords with at least one occurrence into the software as the unit of analysis. These results are shown in the network view.

After analyzing the metrics, we completely read the articles to extract important information. This endeavor allowed us to map knowledge about the therapeutic use of amitriptyline. We generated a graph showing the therapeutic use of amitriptyline over time.

### 4.5. Content Analysis

After selection, the articles were read in full, and information was extracted regarding their content, study design, when they were published, which country the corresponding authors belong to, and the application of the use of amitriptyline over the years in the selected documents. Studies were classified by type based on work by Higgins et al. [107]. This classification is based on the Cochrane Collaboration Glossary: in vivo, in vitro, literature review, systematic review, meta-analysis, observational, and randomized clinical trials studies.

## 5. Conclusions

Our review is the first to report bibliometric characteristics and knowledge mapping for the use of amitriptyline. We also reported, through the analysis of the articles in this top 100, that there was a therapeutic repositioning of amitriptyline. From the early 1960s to the early 1980s, this drug was mostly used to treat depressive disorders, but since the early 1980s, it has been used to treat other conditions such as headache, fibromyalgia, neuropathic pain, gastrointestinal disorders, and other types of painful diseases.

## Figures and Tables

**Table 1:** The 100 most-cited articles on amitriptyline.

Rank	Authors	Number of Citations			Main Findings	Journal	Impact Factor
		Web of Science—Core Collection	Scopus	Google Scholar			
1	Steru et al., 1985 [8]	2671	2711	3902	Antidepressant drugs, including amitriptyline, shorten the duration of immobility in an in vivo mouse study.	Psychopharmacology	3.4
2	Cipriani et al., 2018 [9]	842	1371	2318	Antidepressants, including amitriptyline, were more effective than placebo in adults with major depressive disorder in this systematic review of randomized controlled trials.	Lancet	168.9
3	Max et al., 1992 [10]	834	1006	1461	In this randomized clinical trial, amitriptyline was observed to reduce pain caused by peripheral nerve disease.	New England Journal of Medicine	158.5
4	Hershman et al., 2014 [11]	789	810	1224	In this systematic review of randomized controlled trials, after analysis, there was no significant difference between groups in terms of symptom-based appearance or progression of chemotherapy-induced peripheral neuropathy.	Journal of Clinical Oncology	45.3
5	Furukawa et al., 2006 [12]	758	970	1206	Randomized clinical trials of amitriptyline failed to provide standard deviations of their continuous outcome measures in depression.	Journal of Clinical Epidemiology	7.2
6	Carlsson et al., 1969 [13]	722	564	777	In this in vivo study, pretreatment with antidepressant drugs, including amitriptyline, largely prevented 5-hydroxytryptamine depletion.	European Journal of Pharmacology	5
7	Monsma et al., 1993 [14]	689	609	883	Amitriptyline acts with high affinity on a serotonin receptor subtype in this in vitro assay.	Molecular Pharmacology	3.6
8	McQuay et al., 1996 [15]	636	819	1279	Antidepressants, including amitriptyline, are effective in relieving neuropathic pain, according to this systematic review.	Pain	7.4
9	Macfarlane et al., 2017 [16]	622	627	1181	Antidepressants, including amitriptyline, are effective in relieving neuropathic pain, according to this literature review.	Annals of the Rheumatic Diseases	27.4
10	Seppi et al., 2011 [17]	595	649	1038	This literature review does not show sufficient evidence of the efficacy of amitriptyline in the treatment of Parkinson’s disease with a focus on non-motor symptoms.	Movement Disorders	8.6
11	Lanquillon et al., 2000 [18]	580	631	885	Patients classified as having a psychopathological outcome responded positively after the use of amitriptyline in this non-randomized clinical trial.	Neuropsychopharmacology	7.6
12	Anderson, 2000 [19]	569	641	1054	This systematic review indicates that selective serotonin reuptake inhibitors have a modest advantage in tolerability over most tricyclic antidepressants, including amitriptyline.	Journal of Affective Disorders	6.6
13	Kelly et al.,1997 [20]	566	597	787	Other behavioral, neurotransmitter, and immunological changes have been shown to be attenuated by antidepressant treatment, particularly with amitriptyline.	Pharmacology & Therapeutics	13.5
14	Silberstein et al., 2012 [21]	551	610	813	Amitriptyline is effective in migraine prevention, according to this study.	Neurology	48
15	Kirchheiner et al., 2004 [22]	551	629	903	Antidepressants, including amitriptyline, can cause CYP2D6 or CYP2C19 gene variation, depending on the dose, according to this literature review.	Molecular Psychiatry	11
16	Radley et al., 2006 [23]	522	563	901	In this cross-sectional study, amitriptyline hydrochloride had, along with others, the highest proportion of off-label use among specific drugs.	Archives Of Internal Medicine	39
17	Carville et al., 2008 [24]	513	591	1078	Studies recommend amitriptyline for the treatment of fibromyalgia syndrome, as it is shown to be effective.	Annals of the Rheumatic Diseases	27.4
18	Max et al., 1987 [25]	507	571	851	Amitriptyline relieves pain in diabetic neuropathy, and this effect is independent of mood elevation.	Neurology	48
19	Evers et al., 2009 [26]	505	539	922	Second-choice drugs for migraine prophylaxis include amitriptyline.	European Journal of Neurology	5.1
20	Heikkila et al., 1975 [27]	484	427	605	All drugs studied, including amitriptyline and non-cocaine, are uptake inhibitors of neurotransmitters and releasers.	Biochemical Pharmacology	5.8
21	Hu et al., 2005 [28]	480	553	860	Amitriptyline had a decreased plasma concentration curve after concomitant ingestion of *Hypericum* extract.	Drugs	11.5
22	Rice et al., 2001 [29]	465	563	842	Amitriptyline, in terms of efficacy and side effects for pain relief in diabetic neuropathy, is similar to gabapentin.	Pain	7.4
23	Kost et al., 1996 [30]	461	548	890	Amitriptyline decreased neuronal reuptake of noradrenaline and serotonin.	New England Journal of Medicine	158.5
24	Carlsson et al., 1969 [13]	458	351	473	Antidepressant agents such as amitriptyline show remarkably weak activity on central noradrenaline receptor neurons.	European Journal of Pharmacology	5
25	Wolf et al., 2008 [31]	457	473	750	Amitriptyline has not shown any benefit in improving neuropathic symptoms.	European Journal of Cancer	8.4
26	Ayata et al., 2006 [32]	449	462	635	Chronic daily administration of dose-dependent migraine prophylactic drugs, including amitriptyline, suppressed the frequency of cortical spreading depression by 40%–80%.	Annals Of Neurology	11.2
27	Katon et al., 1992 [33]	448	512	694	Only 20% of patients prescribed first-generation antidepressants, including amitriptyline, experienced clinical improvement.	Medical Care	3
28	Teichgraeber et al., 2008 [34]	445	455	585	Amitriptyline normalizes pulmonary ceramide levels and prevents all pathologic findings, including susceptibility to infection, by blocking acid sphingomyelinase.	Nature Medicine	82.9
29	Rasquin-Weber et al., 1999 [35]	443	623	989	Daily treatment of gastrointestinal disorders with amitriptyline may reduce the frequency or eliminate episodes.	Gut	24.5
30	Snyder et al., 1977 [36]	440	321	516	Amitriptyline hydrochloride is about 10 times more potent than imipramine hydrochloride in its binding potency to the brain and gut muscarinic acetylcholine receptors.	Archives of General Psychiatry	25.8
31	Jick et al., 2004 [37]	438	504	761	There were no significant associations between use of a specific antidepressant, including amitriptyline, and risk of suicide.	Jama-Journal of the American Medical Association	120.7
32	Benfield et al., 1986 [38]	432	410	533	Amitriptyline is more effective in relieving sleep disturbances in depressed patients compared to fluoxetine.	Drugs	11.5
33	Gillman et al., 2007 [39]	421	468	703	Amitriptyline is possibly less effective than nortriptyline for migraine and pain syndromes.	British Journal of Pharmacology	7.3
34	Horn et al., 1971 [40]	420	314	448	*N*-demethylation of amitriptyline reduces inhibition of hypothalamic catecholamine uptake 24-fold.	Molecular Pharmacology	3.6
35	Onghena et al., 1992 [41]	415	498	742	The average patient with chronic pain who received an antidepressant treatment, including amitriptyline, had less pain than 74% of patients with chronic pain who received a placebo.	Pain	7.4
36	Watson et al., 1982 [42]	414	489	738	Amitriptyline is useful to treat postherpetic neuralgia (PHN) and may not act as an antidepressant.	Neurology	48
37	Maas, 1975 [43]	409	266	539	Patients who excrete normal to greater than normal amounts of urinary 3-methoxy-4-hydroxyphenylethyleneglycol have a favorable response to treatment with amitriptyline.	Archives of General Psychiatry	25.8
38	Ford et al., 2009 [44]	399	462	778	Antidepressants including amitriptyline are effective in treating irritable bowel syndrome.	Gut	24.5
39	Mills et al., 1968 [45]	388	214	441	Amitriptyline inhibits the second phase of aggregation at concentrations below those that interfere with adenosine diphosphate (ADP) aggregation.	Journal Of Physiology-London	5.5
40	Mynors-Wallis et al., 1995 [46]	384	437	649	Amitriptyline is effective, feasible, and acceptable in six sessions of treatment for problems associated with major depression.	Bmj-British Medical Journal	105.7
41	Klerman et al., 1974 [47]	384	344	754	Patients who received amitriptyline and little psychotherapy had a 12% relapse rate compared with a 16% rate for those who received more psychotherapy and no medication.	American Journal of Psychiatry	17.7
42	Saarto et al., 2010 [48]	379	454	1182	It remains unclear whether antidepressants such as amitriptyline prevent the development of neuropathic pain (preventive use).	Cochrane Database of Systematic Reviews	8.4
43	Sanchez et al., 1999 [49]	376	390	544	Amitriptyline has similar in vitro reuptake inhibitory potential for 5-hydroxytryptamine and norepinephrine.	Cellular And Molecular Neurobiology	4
44	Bendtsen, 2000 [50]	374	441	767	Amitriptyline reduced headaches significantly more than placebo.	Cephalalgia	4.9
45	Richelson et al., 2000 [51]	369	323	431	Amitriptyline (Kd = 18 nM) is less potent than trazodone (Kd = 324 µM) at the muscarinic receptor.	Journal of Pharmacology and Experimental Therapeutics	3.5
46	Kaptchuk et al., 2006 [52]	368	399	630	In patients with persistent arm pain, 25 mg of amitriptyline produced a steady-state blood concentration for 4 weeks.	Bmj-British Medical Journal	105.7
47	Paykel et al., 1999 [53]	365	423	710	Amitriptyline reduces relapse and recurrence rates compared to placebo in residual depression.	Archives of General Psychiatry	25.8
48	Cipriani et al., 2016 [54]	365	335	522	Amitriptyline is less effective than fluoxetine in major depressive disorder in children and adolescents.	Lancet	168.9
49	Serretti et al., 2010 [55]	363	402	655	Amitriptyline was linked to a higher risk of weight gain.	Journal of Clinical Psychiatry	5.3
50	Anttila et al., 2001 [56]	363	395	646	In major depression, amitriptyline has similar efficacy to mirtazapine.	Cns Drug Reviews	Discontinued
51	Jackson et al., 2000 [57]	355	472	721	Amitriptyline appears to be effective in treating functional gastrointestinal disorders.	American Journal of Medicine	5.9
52	Goldenberg et al., 1986 [58]	354	369	650	Amitriptyline, or amitriptyline and naproxen, is an effective therapeutic regimen for patients with fibromyalgia.	Arthritis And Rheumatism	5
53	Braithwa et al., 1972 [59]	344	194	329	Patients who had plasma amitriptyline concentrations (<120 ng/mL) had a poor clinical response.	Lancet	168.9
54	Spiker et al., 1986 [60]	336	308	472	In delusional depression, the combination of amitriptyline and perphenazine was superior (0.01) to amitriptyline alone.	European Journal of Pharmacology	5
55	Ross et al., 1969 [61]	336	283	382	In vitro, amitriptyline was more active than its secondary analog, nortriptyline, in inhibiting 5-hydroxytryptamine uptake in vitro.	American Journal of Psychiatry	17.7
56	Nguyen, 2001 [62]	333	341	483	Amitriptyline has a high affinity for the histamine H1 receptor.	Molecular Pharmacology	3.6
57	Kirchherr et al., 2006 [63]	332	351	459	Amitriptyline can be used for simultaneous determination at low concentrations based on high-performance liquid chromatography.	Journal of Chromatography B-Analytical Technologies in the Biomedical and Life Sciences	3
58	Miyasaki et al., 2006 [64]	330	446	658	Amitriptyline may be considered to treat depression in Parkinson’s disease without dementia (level of evidence: C).	Neurology	48
59	Reul et al.,1993 [65]	326	340	398	There is an increase in amitriptyline on limbic magnetic resonance imaging, a decrease in adrenal size, and upregulation of glucocorticoid receptor (GR) in particular brain regions.	Endocrinology	4.8
60	Bulik et al., 2007 [66]	324	392	724	Amitriptyline is associated with better treatment efficacy compared with cyproheptadine in anorexia nervosa.	Archives of General Psychiatry	25.8
61	Bielski et al., 1976 [67]	324	249	489	In this study, amitriptyline was divided into predictors of good and poor response. Criteria such as high socioeconomic class and delusions were considered.	International Journal of Eating Disorders	5.5
62	Ueng et al., 1997 [68]	319	387	488	Amitriptyline has positive cooperativity in the oxidation of various substrates in systems containing purified recombinant CYP3A4 from bacteria.	Biochemistry	2.9
63	O’Connor, 2009 [69]	318	326	515	Amitriptyline proves to be cost-effective or dominant over other neuropathic pain strategies.	Pharmacoeconomics	4.4
64	Heninger et al.,1984 [70]	318	256	349	There was an increased antidepressant effect in patients treated with amitriptyline hydrochloride for refractory depression.	Archives of General Psychiatry	25.8
65	Hicks et al., 2013 [71]	314	339	478	Patients taking amitriptyline in combination with a potent CYP2D6 inhibitor such as fluoxetine may experience dramatic increases in amitriptyline plasma concentrations.	Clinical Pharmacology and Therapeutics	6.7
66	Montigny et al., 1983 [72]	313	241	353	Animal studies have shown that tricyclic antidepressants such as amitriptyline sensitize forebrain neurons to serotonin.	Pharmacology Biochemistry and Behavior	3.6
67	Sherman et al., 1982 [73]	313	303	445	Chronic administration of amitriptyline effectively reversed learned helplessness in depression.	Archives of General Psychiatry	25.8
68	Ray et al., 1992 [74]	309	378	568	Amitriptyline increases the relative risk of traffic accidents in older adults receiving a dose of ≥125 mg.	American Journal of Epidemiology	5
69	Chew et al., 2008 [75]	307	356	562	At typical doses administered to older adults, amitriptyline demonstrated an AA > 15 pmol/mL.	Journal of the American Geriatrics Society	6.3
70	Pond et al., 1984 [76]	305	271	449	Sometimes amitriptyline is only detected in plasma after an oral dose.	Clinical Pharmacokinetics	4.5
71	Thapa et al., 1998 [77]	304	367	542	There was little difference in rates of falls between those treated with tricyclic antidepressants, including amitriptyline, and those treated with selective serotonin reuptake inhibitors.	New England Journal of Medicine	158.5
72	Goldenberg et al., 1996 [78]	301	396	671	Amitriptyline and fluoxetine are effective for fibromyalgia and work better in combination than either drug alone.	Arthritis and Rheumatism	5
73	Mann, 2005 [79]	300	338	628	Amitriptyline inhibits the reuptake of serotonin and norepinephrine.	International Journal of Pharmaceutics	5.8
74	Nii & Ishii, 2005 [80]	300	253	365	Amitriptyline hydrochloride resulted in a slightly higher encapsulation efficiency when dissolved in water than chloroform.	New England Journal of Medicine	158.5
75	Morris et al., 1974 [81]	296	221	498	Tricyclic antidepressants, including amitriptyline, are more effective than placebo in treating depression.	Archives of General Psychiatry	25.8
76	Max et al., 1988 [82]	295	355	479	Greater relief was associated with higher doses of amitriptyline, up to a maximum dose of 150 mg/day in PHN neuralgia.	Psychopharmacology	3.4
77	Bodnoff et al., 1988 [83]	295	305	414	Chronic but non-acute injections of amitriptyline significantly reduced the latency to start eating compared with controls.	Neurology	48
78	Gardiner et al., 2006 [84]	294	343	526	Patients with a dysfunctional CYP2D6 allele had an increased risk of side effects with amitriptyline.	Current Pharmaceutical Design	3.1
79	Pacher et al., 2005 [85]	294	319	491	Amitriptyline inhibits cardiovascular calcium, sodium, and potassium channels, often leading to life-threatening arrhythmias.	Pharmacological Reviews	21.1
80	Arnold et al., 2000 [86]	292	393	637	Amitriptyline significantly reduced the severity and the number of tender points in patients with fibromyalgia.	Psychosomatics	22.8
81	Fava, 2000 [87]	291	323	574	Weight gain during antidepressant treatment, including with amitriptyline, may be a sign of improvement in patients who have weight loss as a symptom of depression or a residual symptom.	Journal of Clinical Psychiatry	5.3
82	Lewis et al., 2002 [88]	290	382	580	Amitriptyline data for migraine in children and adolescents were insufficient.	Neurology	48
83	Bril et al., 2011 [89]	284	429	852	Amitriptyline is likely to be effective and should be considered for the treatment of painful diabetic neuropathy (level of evidence: B).	Neurology	48
84	Morello et al., 1999 [90]	283	387	583	There is no significant difference between amitriptyline and gabapentin in the treatment of diabetic neuropathic pain.	Archives of Internal Medicine	39
85	Duby et al., 2004 [91]	274	301	616	The use of amitriptyline as evidenced in clinical trials is supported for the management of symptoms of diabetic neuropathy.	Journal of Clinical Pharmacology	2.9
86	Venkatakrishnan et al., 2001 [92]	274	278	425	Amitriptyline demethylation was inhibited by S-mephenytoin (500 µM) in livers lacking CYP2C19.	American Journal of Health-System Pharmacy	2.7
87	Leijon et al., 1989 [93]	272	334	488	Amitriptyline produced a statistically significant reduction in pain compared with placebo.	Pain	7.4
88	Linkowski et al., 1987 [94]	271	279	382	Treatment with amitriptyline and electroconvulsive therapy corrected neuroendocrine abnormalities.	Journal of Clinical Endocrinology & Metabolism	5.8
89	Frahnert et al., 2003 [95]	267	289	347	Serum or plasma samples for therapeutic drug monitoring of about 30 antidepressants, including amitriptyline based on high-performance liquid chromatography.	Journal of Chromatography B-Analytical Technologies in the Biomedical and Life Sciences	3
90	Reimherr et al., 1990 [96]	267	261	337	The amitriptyline treatment group showed significantly greater improvement compared to the placebo group in this randomized clinical trial.	Journal of Clinical Psychiatry	5.3
91	Holroyd et al., 2001 [97]	265	356	569	Antidepressant medications including amitriptyline and stress management therapy are modestly effective in treating chronic tension-type headaches.	Jama-Journal of the American Medical Association	120.7
92	Thomas et al., 1987 [98]	264	265	316	Amitriptyline was 15 times stronger than paroxetine in muscarinic receptor affinity in this in vivo animal study.	Psychopharmacology	3.4
93	Field et al., 1999 [99]	263	284	352	Amitriptyline has a lower antiallodynic profile than gabapentin for neuropathic pain, as evidenced in this in vivo animal study.	Archives of General Psychiatry	25.8
94	Beckmann et al., 1975 [100]	263	160	332	Amitriptyline treatment was associated with a significant decrease in 3-methoxy-4-hydroxyphenylethylene glycol excretion seen in this cross-sectional study.	Pain	7.4
95	Green et al., 1977 [101]	259	181	269	Chemically similar tricyclic antidepressants, including amitriptyline, can also have this competitive histamine H2 antagonist activity via in vivo techniques.	Nature	64.8
96	Guyatt et al., 1990 [102]	256	273	415	This study reviewed the feasibility of randomized clinical trials, and in some of these trials, it was observed that amitriptyline was not effective for fibrositis, for example.	Annals of Internal Medicine	39
97	Paykel et al., 1988 [103]	256	248	345	The results of this randomized clinical trial indicate that tricyclic antidepressants, including amitriptyline, are of considerable benefit for major depression compared to placebo.	Journal of Affective Disorders	6.6
98	Davidson et al., 1990 [104]	253	302	539	In this randomized clinical trial in 56 veterans with chronic post-traumatic stress disorder, it was observed that, at the end of treatment, 64% of patients treated with amitriptyline and 72% of patients treated with placebo samples still met diagnostic criteria for post-traumatic stress.	Archives of General Psychiatry	25.8
99	Pancrazio et al., 1998 [105]	251	283	357	This in vitro study demonstrates that the onset of amitriptyline’s effects on inhibiting neuronal sodium channels was significantly faster than that of fluoxetine.	Journal of Pharmacology and Experimental Therapeutics	3.5
100	Lidbrink et al., 1971 [106]	245	182	252	In this in vivo study, it was observed in albino male Sprague-Dawley rats that amitriptyline preferentially blocks 5-hydroxytryptamine receptors in central 5-hydroxytryptamine neurons.	Neuropharmacology	4.7

**Table 2:** Publication periods of the 100 most-cited articles on amitriptyline.

Period of Publication	Number of Articles WoS Core Collection	Number of Citations WoS Core Collection	Citation Density
1968–1978	15	5772	384.80
1979–1988	16	7713	482.06
1989–1998	18	7291	405.06
1999–2008	39	14,984	384.21
2009–2018	12	5947	495.58

**Table 3:** Study types of the 100 most-cited articles on amitriptyline.

Study Design	Number of Articles	Number of Citations
Laboratorial study		
In vivo study—animal	15	8116
In vitro	9	3317
In vivo and in vitro	1	245
Literature review	31	11,968
Systematic review	13	6162
Guideline	2	772
Cohort	2	613
Case control	1	438
Methodological study	1	758
Cross-sectional study	4	1617
Randomized clinical trial	18	6537
Non-randomized clinical trial	3	1164

**Table 4:** Web of Science (WoS) search strategy.

Search Key
(amitriptyline or tryptine or amitrip or amitrol or tryptanol or tryptizol or triptafen or sarotex or domical or laroxyl or lentizol or amitriptylin or amineurin or anapsique or damilen or endep or novoprotect or “amitriptylin rph” or syneudon or saroten or elavil or desitin or damitriptyline or “amitriptylin beta” or “amitriptylin desitin” or “amitriptylin neuraxpharm” or “amitriptyline hydrochoride”)

## Data Availability

Data is contained within the article.
